# Drug-repositioning screening identified fludarabine and risedronic acid as potential therapeutic compounds for malignant pleural mesothelioma

**DOI:** 10.1007/s10637-020-01040-y

**Published:** 2020-12-09

**Authors:** Irene Dell’Anno, Sarah A. Martin, Marcella Barbarino, Alessandra Melani, Roberto Silvestri, Maria Bottaro, Elisa Paolicchi, Alda Corrado, Monica Cipollini, Ombretta Melaiu, Antonio Giordano, Luca Luzzi, Federica Gemignani, Stefano Landi

**Affiliations:** 1 Department of Biology, Genetic Unit, University of Pisa, 56126 Pisa, Italy; 2 Centre for Cancer Cell and Molecular Biology, Barts Cancer Institute, Queen Mary University of London, Charterhouse Square, London, EC1M 6BQ UK; 3 Department of Medical Biotechnologies, University of Siena, 53100 Siena, Italy; 4 Sbarro Institute for Cancer Research and Molecular Medicine, Center for Biotechnology, College of Science and Technology, Temple University, Philadelphia, PA 19122 USA; 5 Immuno-Oncology Laboratory, Department of Paediatric Haematology/Oncology and of Cell and Gene Therapy, Ospedale Pediatrico Bambino Gesù, IRCCS, 00165 Rome, Italy; 6 Department of Medicine, Surgery and Neurosciences, Siena University Hospital, 53100 Siena, Italy

**Keywords:** Mesothelioma, Drug repositioning, Fludarabine, Antimetabolite, Risedronic acid, Bisphosphonate

## Abstract

*Objectives* Malignant pleural mesothelioma (MPM) is an occupational disease mainly due to asbestos exposure. Effective therapies for MPM are lacking, making this tumour type a fatal disease. *Materials and Methods* In order to meet this need and in view of a future “drug repositioning” approach, here we screened five MPM (Mero-14, Mero-25, IST-Mes2, NCI-H28 and MSTO-211H) and one SV40-immortalized mesothelial cell line (MeT-5A) as a non-malignant model, with a library of 1170 FDA-approved drugs. *Results* Among several potential compounds, we found that fludarabine (F-araA) and, to a lesser extent, risedronic acid (RIS) were cytotoxic in MPM cells, in comparison to the non-malignant Met-5A cells. In particular, F-araA reduced the proliferation and the colony formation ability of the MPM malignant cells, in comparison to the non-malignant control cells, as demonstrated by proliferation and colony formation assays, in addition to measurement of the phospho-ERK/total-ERK ratio. We have shown that the response to F-araA was not dependent upon the expression of DCK and NT5E enzymes, nor upon their functional polymorphisms (rs11544786 and rs2295890, respectively). *Conclusion* This drug repositioning screening approach has identified that F-araA could be therapeutically active against MPM cells, in addition to other tumour types, by inhibiting STAT1 expression and nucleic acids synthesis. Further experiments are required to fully investigate this.

**Supplementary Information:**

The online version contains supplementary material available at 10.1007/s10637-020-01040-y.

## Introduction

The research and development for releasing new chemical entities into the therapeutic market is a complex, time-consuming, and costly process. An alternative approach could be by using “drug repositioning” (DR) approach, consisting of the identification of new uses for existing and approved drugs. DR could allow by-passing the pre-clinical development and the early phases of clinical experimentation, given that the safety profile, the pharmacokinetic and pharmacodynamic properties are already well-defined. As showed by Van der Velden and co-workers, this type of approach could facilitate and accelerate the translation of existing drugs into the clinical use as novel anticancer agents [1]. DR would be even more appropriate for the so-called “orphan diseases”, i.e. rare conditions where there is limited commercial interest for investing in the research and development of novel compounds.

Because of its rarity (1.6–3.0 cases for 100,000 in the western countries, WHO) [2], Malignant Pleural Mesothelioma (MPM) is a disease that would benefit from such a strategy. Mesothelioma is an insidious neoplasm arising mainly from pleura (65%–70%), although other mesothelial surfaces as peritoneum, tunica vaginalis of the testis, and pericardium have been documented [3]. Currently, first-line chemotherapy for MPM consists of a combination of cisplatin with either pemetrexed or raltitrexed, where cisplatin could be substituted with carboplatin in elderly patients [4, 5]. However, despite innovative approaches, including multimodality treatments [6, 7], targeted therapies (e.g. bevacizumab), or immunotherapy, an effective cure is still lacking, and the prognosis remains poor. Since there are no effective second-line options and other chemotherapeutic agents, such as gemcitabine and vinorelbine, showed only limited benefit, the average overall survival is 18 months from diagnosis [2]. In summary, a DR approach could help to provide a better therapeutic strategy for MPM patients, which is supported by the recent repurposing of the pyrvinium, pamoate [8]. Thus, to this end, in the present work we carried out a study where 1170 FDA-approved drugs were assayed for their cytotoxic effects on a panel of MPM cell lines.

## Materials and methods

### Mesothelioma cell lines

In this work, we employed nine commercially available immortalized cell lines (the malignant Mero-14, Mero-25, IST-Mes2, NCI-H28, MSTO-211H, Mero-41, Ren, and the non-malignant MeT-5A and LP-9) and three primary cultures (two from patients MMP1, MMP2, and one from a pneumothorax HMC7) locally grown from surgically resected specimens. MeT-5A and Mero-41 were purchased from ATCC, whereas LP-9 cells were from Coriell Institute (Camden, New Jersey, USA). Mero-14, Mero-25, IST-Mes2, and Ren were kindly donated by Istituto Tumori of Genova (Italy), NCI-H28 cells by collaborators of the Pharmaceutical Department of University of Pisa, and finally, MSTO-211H by collaborators of the Barts Cancer Institute (London, UK). MeT-5A, originally derived from normal mesothelial cells and immortalized with SV-40, were grown in Medium 199 with HEPES (Life Technologies, Carlsbad, CA, USA) supplemented with 3.3 nM epidermal growth factor (EGF, Life Technologies), 400 nM hydrocortisone (Sigma Aldrich), and 870 nM insulin (Life Technologies). Mero-14, Mero-25, IST-Mes2, and Ren were cultured in DMEM medium (Euroclone, Milan, Italy), Mero-41 in HAM’S F10 (Euroclone) supplemented with 2 mmol/L l-glutamine (Euroclone), whereas NCI-H28 and MSTO-211H in RPMI 1640 (Euroclone). All media were supplemented with 1% penicillin-streptomycin (Euroclone) and 10% of FBS (Euroclone), with the exception of Mero-41 requiring a supplementation of 15% of FBS. HMC7 cells were isolated from a patient with a bullous emphysema in a spontaneous pneumothorax. MMP1 and MMP2 mesothelioma cell lines were isolated from patients’ who underwent surgery at the Thoracic Surgery Unit (Siena, Italy) for decortication, without prior chemotherapy or radiotherapy [9]. All specimens were collected from patients diagnosed for pleural mesothelioma (MMP1: epithelioid; MMP2: biphasic) with their written consent. The study was approved by the Research Ethics Committee (Comitato Etico Regione Toscana-Area Vasta Sud Est; #CCMESOLUNG). LP-9, HMC7, MMP1 and MMP2 cell lines were cultured in Medium 199 (Euroclone), supplemented with 2 mmol/L l-glutamine (Euroclone), 100 U/mL penicillin, 100 μg/mL streptomycin (Euroclone), 10% FBS (Euroclone), 20 ng/mL hEGF (Sigma-Aldrich), 0.4 μg/ml hydrocortisone (Sigma-Aldrich). All cell lines, grown at 37 °C and 5% CO_2_, were routinely passaged every 1–2 weeks.

### Compound library screen

The FDA-approved compound library incorporating 1170 drugs was purchased from Selleck Chemicals (Houston, TX, USA). Cells were plated in 96-well plates, at different concentrations, and treated with vehicle [0.01% dimethyl sulfoxide (DMSO)] or the compound library (average compound concentration of the library was 10 μM). After 4 days of incubation with the drug, cell viability was assessed using the CellTiter-Glo® assay (Promega, Madison, WI, USA) according to the manufacturer’s instructions. Drug screening was performed in duplicate.

### Validation experiments (cell titer assay)

For validation experiments, cells were plated in 96-well plates and treated with F-araA or RIS (or related compounds, see below) at 24 and 72 h after seeding, with a range of concentrations (0.1 μM, 1 μM, 10 μM and 100 μM). Cell viability was assayed after 5 days using the CellTiter-Glo® assay (Promega) according to the manufacturer’s instructions. Each drug was validated in triplicate. Stock solutions of the drug were prepared in DMSO (Euroclone), PBS (Euroclone), or sterile water, according to their respective solubility. Three independent validation experiments were performed, each in triplicate.

### Chemicals

Alexidina, Alendronate, Cladribine, Clofarabine, Carmofur, Erlotinib, Ibandronate, Trifluothymidine and ATP were purchased from Caymann Chemicals (Ann Arbor, MI, USA). 6-Thioguanine, Cytarabine, 6-MercaptoPurine and Zoledronate were purchased from Sigma Aldrich (Saint Louis, MO, USA). Gemcitabine was purchased from Chemodex, Fludarabine from AdooQ® Bioscience (Irvine, CA, USA), Risedronic Acid from LKT Laboratories (St Paul, MN USA), Cisplatin from AdipoGen® Life Science (Liestal, Switzerland).

### MTT (3-(4, 5-dimethylthiazolyl-2)-2,5-diphenyltetrazolium bromide) assay

After seeding in 96-well plates, cells were incubated for 24 h and treated with either the vehicle or the drug at the indicated concentrations (1 and 10 μM). Proliferation was assessed at different time points (0, 24, 48 and 72 h after treatment) by adding MTT solution (5 mg/mL, solved in PBS) (Sigma Aldrich) in each well (incubation time: 3 h at 37 °C). Upon removal of culture medium, MTT crystals were dissolved in 50 μL of DMSO and absorbance at 595 and 655 nm was measured using the Bio-Rad Imark microplate reader (Bio-Rad, Hercules, CA, USA). Three independent experiments, each in triplicate, were performed. For combination studies with cisplatin, primary cell lines were seeded 24 h before treatment with the drugs, alone and in combination, and then incubated for further 72 h. Control cells were treated with the same amount of vehicle used to deliver the molecules. All the experiments were carried out in duplicate with at least four replicates for each experiment.

### Colony formation “CF” assay

Cells, seeded in six-well plates, were exposed to the vehicle or the drug at the indicated concentration (1 and 10 μM). Cells were treated every 3 days, removing the medium and replacing it with fresh medium containing the drug. After 10 days, cells were fixed and stained with Sulphorodamine B (SRB). Three independent experiments were performed.

### Caspase 3/7 activation

Detection of active caspases 3/7, as marker of apoptosis, occurred through Caspase-Glo® 3/7 Assay (Promega). Cells were seeded in a six-well plate and treated with F-araA twice, one and 3 days after seeding. The day of detection of caspases activity, cells were collected by trypsinization and approximately 15 × 10^3^ cells were transferred in a 96-well white plate (Corning, Corning, NY, USA). An equal volume of Caspase 3/7-Glo reagent was added in each well and the samples were incubated at constant room temperature for 1 h. Luminescence was detected with FLUOstar® Omega microplate reader (BMG LabTech, Offenburg, Germany). Three independent experiments were performed, each in triplicate.

### Genotyping

Genomic DNA was isolated from cells using PureLink™ Genomic DNA Mini Kit (Thermo Fisher), according to the manufacturer’s protocol. Genetic variants in *NT5E* (rs2295890) and *DCK* (rs11544786) were screened using PCR followed by sequencing. PCR was carried out using 100 ng of DNA Genomic as template and Q5® High-Fidelity DNA Polymerase (New England Biolabs, Ipswich, MA, USA), assembling all reaction components in a final volume of 25 μL according to the manufacturer’s protocol. Thermocycling Conditions were 30 s at 98 °C, 34 cycles at 98 °C for 10 s, Tm°C for 20 s and 72 °C for 30 s, with a final extension at 72 °C for 2 min. The amplified products were analysed for their size by electrophoresis on a 2% agarose gel stained with ethidium bromide and then purified using ExpinTM Combo GP (GeneAll, Seoul South Korea), according to the manufacturer’s protocol. For *DCK*, we employed the following primers: PCR: F: AGCCCTATTGACCATTAATTTTG, R: ACGTACAAGCCATTTATACATAC, Tm = 61 °C; Sequencing: F: GCACATTCAAAATAGGAACTTACA, R: ACGTACAAGCCATTTATACATAC. For *NT5E*, we employed the following primers: PCR: F: TCTCAACCCAACAGGAAGCG, R: CAGGAAGAGTGGAGAGGTTGTT, Tm = 67 °C; Sequencing: F: CCTCTCTTTACTCCTCCTCTCT, R: GTTCGGCTGCTGGTGCG.

### Protein extraction and Western blot

For protein extraction and western blots, cell pellets were suspended in ICE-cold RIPA buffer, containing PMSF, protease and phosphatase inhibitor (all purchased from Sigma Aldrich). After 15 min of ice-incubation the extracts were clarified by centrifugation at 16000 g for 15 min, at 4 °C. The protein concentration was determined with BCA Total protein colorimetric assay QuantumProtein (Euroclone). Lysates were incubated with 4x Laemmli Sample Buffer (Bio-Rad), according to the manufacturer instructions, for 10 min at 99 °C. An amount of 10 μg of proteins for each sample was loaded onto 10–15% SDS polyacrylamide gel (Thermo Fisher Scientific and Bio-Rad). Proteins were transferred with Trans-Blot® Turbo™ Transfer System (Bio-Rad) onto PVDF membrane (Bio-Rad). Running Buffer was purchased from Bio-Rad. The membrane was blocked for 1 h with 5% *w*/*v* non-fat dry milk in TBS buffer (Bio-Rad) containing 0.05% Tween 20 (Sigma Aldrich), washed, and successively incubated with different primary antibodies at 4 °C overnight. The membranes were washed three times for 8 min and incubated with the HRP-conjugated secondary antibody for 1 h at room temperature. After washing, three times for 8 min, the blot was exposed to Clarity or Clarity Max ECL Western Blotting Substrate (Bio-Rad) and the image was acquired with Chemidoc MP (Bio-Rad). The following primary and secondary antibodies, purchased from Proteintech Europe (Manchester, UK), were employed: DCK (17758–1-AP DCK Rabbit Polyclonal antibody), NT5E (12231–1-AP NT5E/CD73 Rabbit Polyclonal antibody,), BAX (50599–2-Ig BAX Rabbit Polyclonal antibody), BCL2 (12789–1-AP BCL2 Rabbit Polyclonal antibody), β-actin (60008–1-Ig beta Actin Mouse Monoclonal antibody), GAPDH (60004–1-Ig Mouse Monoclonal antibody), Peroxidase-conjugated Affinipure Goat Anti-Mouse IgG(H + L) (SA00001–1) and Peroxidase-conjugated Affinipure Goat Anti-Rabbit IgG(H + L) (SA00001–2). We bought from Cell Signaling Technology (Danvers, MA, USA) the primary antibodies for STAT1 (#9172, Polyclonal Rabbit), Erk1/2 (#4695 T, p44/42 MAPK (Erk1/2) (137F5) Monoclonal Rabbit), phospho-Erk1/2 (#4370S, Phospho-p44/42 MAPK (Erk1/2) (Thr202/Tyr204) (D13.14.4E) XP® Monoclonal Rabbit).

### Restoring of dNTP pool

After seeding in 96-well plates, cells were incubated for 24 h and then treated with either vehicle, 1 μM of F-araA, 50 μM of ATP, alone or in combination. Proliferation was assessed 72 h after treatment by adding MTT solution, and following the procedure previously described. Three independent experiments were performed, each in duplicate.

### Statistical analysis

Luminescence readings from each well were log-transformed and normalized according to the median signal on each plate and then standardized by use of a Z-score statistic, using the median absolute deviation to estimate the variation in each screen. Z-scores values less than −1.96 (corresponding to the nominal probability level of 2.5% in a two-tails test) were used to select compounds causing a loss of viability in the employed cell lines. The differential activity between MPM and MeT-5A cell lines was calculated as the difference between the average of Z-scores of all MPM cell lines and that of MeT-5A, and it is now on indicated as “∆-act”. In order to focus our experimentation on the most interesting molecules, drugs were ranked according to ∆-act. Then, among the statistically significant ones, we discharged the ones already assayed in clinical trials on MPM and we picked the remaining two at the top of the list (namely F-araA and RIS). Other potentially interesting molecules (placed lower in the rank) could be assayed in further experimentations. For the validation experiments, unless otherwise stated, data represent standard error of the mean of three independent experiments. The two-tailed paired Student’s t test was used to determine statistically significance with *p* < 0.05 regarded as significant. Data analysis and summary graphs were produced with the software GraphPad Prism 7 (San Diego, CA, USA). The cytotoxic interaction between F-araA or RIS and cisplatin was analysed by the software CalcuSyn and expressed as combination index (CI). The CI value enables the quantitative definition of additivity (CI = 1), synergism (CI < 1) or antagonism (CI > 1).

## Results

### Drug repositioning screen of FDA-approved drugs on a panel of MPM cells

To identify compounds active against MPM cells, we carried out a drug repositioning screen of 1170 FDA-approved drugs, at the unique dose of 10 μM, on a panel of five MPM cell lines (Mero-14, Mero-25, IST-Mes2, NCI-H28, and MSTO-211H) and one non-malignant immortalized mesothelial cell line (MeT-5A). Cell viability was assessed using the CellTiter-Glo® assay. Among the 28 compounds showing cytotoxicity on at least three MPM cell lines and not active on MeT-5A cells, we excluded those already assayed in previous clinical trials on MPM (i.e. erlotinib, imatinib mesylate, and sunitinib malate) and we focused on fludarabine (F-araA) and risedronic acid (RIS). Significantly, these drugs showed the highest differential activity of the normalized Z-scores (∆-act) between MPM cell lines and the MeT-5A cells. An overview of these results is reported in Supplemental Table S1 and Supplementary references.

### Viability assay of F-araA and RIS in MPM cell lines and MeT-5A cells

**Fig. 1:**
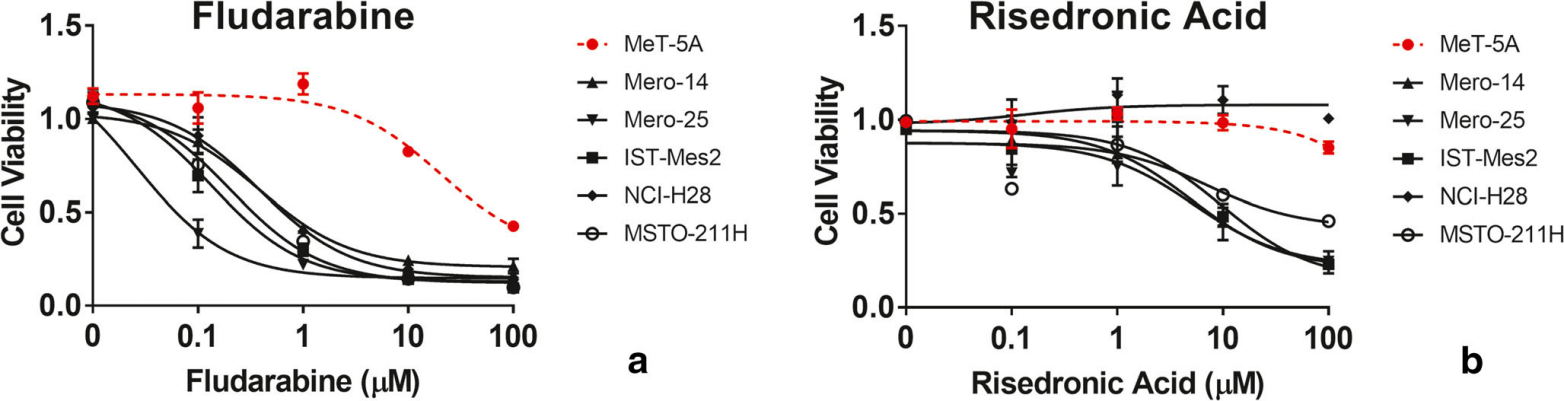
**Response of MPM and MeT-5A cell lines to fludarabine (F-araA) and risedronic acid (RIS)** MPM- and MeT-5A cells (dotted red line) were treated with increasing concentrations (0.1 μM, 1 μM, 10 μM and 100 μM) of **(A)** F-araA and **(B)** RIS. Cell viability was measured after four days treatment using an ATP-based luminescence assay. Data represent mean ± SEM of three independent experiments, each performed in triplicate

To further investigate the potential of F-araA and RIS as novel therapies for MPM, we performed additional cell viability assays using a range of concentrations (between 0.1 μM and 100 μM) for both drugs on the same panel of cell lines used for the original screen [Fig. 1]. For F-araA the difference between MPM cells and MeT-5A was statistically significant at 0.1 μM (*P* < 0.001 for Mero-25, IST-Mes-2, and MSTO-211H; *P* = 0.02 for Mero-14) and maximal at 1 μM (P < 0.001 for all MPM cell lines) [Fig. 1A]. The EC50s were 20.31 μM for MeT-5A in comparison to significantly lower EC50s, 0.39 μM, 0.03 μM, 0.13 μM, 0.38 μM, and 0.20 μM for Mero-14, Mero-25, IST-Mes-2, NCI-H28, and MSTO-211H, respectively. Decreased viability of MeT-5A cells was observed at 10 μM F-araA.

We observed that RIS was less cytotoxic than F-araA in our panel of MPM cell lines [Fig. 1B]. MeT-5A and NCI-H28 displayed a resistance to RIS across the full range of concentrations analysed (up to 100 μM), whereas the remaining MPM cell lines showed a reduced viability of about 55% at 10 μM (P < 0.001) and 75% at 100 μM (*P* < 0.0001). The EC50s were 46.26 μM, 5.01 μM, 5.46 μM, 9.30 μM, and 7.12 μM for MeT-5A, Mero-14, Mero-25, IST-Mes-2, and MSTO-211H, respectively.

To further investigate our findings, we next evaluated additional analogues of F-araA and RIS in our MPM cells. Thus, we performed similar cell viability assays as before, using known antimetabolites (base analogues) sharing similar mechanisms of action with F-araA which included carmofur, cladribine, clofarabine, and trifluorothymidine (Supplemental Figs. S2 & S3). Moreover, we assayed other bisphosphonates (BPs), which are analogues of RIS, including alendronic and ibandronic acids. We also analysed oxethazaine in our cell viability assays due to its shared mechanism of action to BPs, including affecting calcium homeostasis. For these assays, we also treated our panel of MPM cells with cytarabine, gemcitabine, and zoledronic acid as a control, because they are known molecules active on MPM cells. All the molecular structures are depicted in Supplemental Fig. S1. All assayed compounds caused a reduced viability of MPM cell lines but to a weaker extent when compared to F-araA and RIS [Supplemental Figs. S2 & S3]. Thus, we concluded that F-araA and RIS showed the least toxicity in the non-malignant MeT-5A cells, with the greatest reduction in MPM cell viability across our panel. However, when we repeated the assay on a further two commercially available MPM cells, namely, Mero-41 and Ren, we observed that only F-araA confirmed its activity (1 μM) in both cell lines. RIS was cytotoxic only at 100 μM in Ren cells [Supplemental Fig. S4].

We further assayed the effects of F-araA and RIS on the viability of two primary cell lines derived from patients who had undergone surgical resection of MPM, named as MMP1 and MMP2. Cell viability was measured at 72 h after treatment with an increasing range of doses of F-araA and RIS, since preliminary tests suggested that these cells were more resistant than commercial ones to these compounds. Thus, doses were increased up to 25 μM for both drugs and the results showed that the MMP1 cell model had a 60% reduced cell viability upon at 12.5 μM F-araA (*P* < 0.001). In addition, RIS treatment reduced the viability of MMP1 cells by approximately 20% at 25 μM, again showing reduced activity as compared to F-araA [Supplemental Fig. S5].

We also observed that F-araA induced cytotoxic effects of the same order of magnitude as cisplatin, a known chemotherapeutic agent used in the cure of MPM and employed here as a control. We employed the software CalcuSyn to quantify the effects (synergism/inhibition) of the combination of F-araA with cisplatin and this was expressed as a combination index (CI). Interestingly, the combination of cisplatin at 0.625 μM with F-araA at 6.25 μM showed a CI = 0.968, indicating that combined F-araA and cisplatin produced synergistic cytotoxic effects. No synergistic effects were seen for RIS [Supplemental Fig. S6A, B].

### Short-term proliferation and colony formation (CF) assays in MPM cell lines and non-malignant mesothelial cells treated with F-araA and RIS

**Fig. 2:**
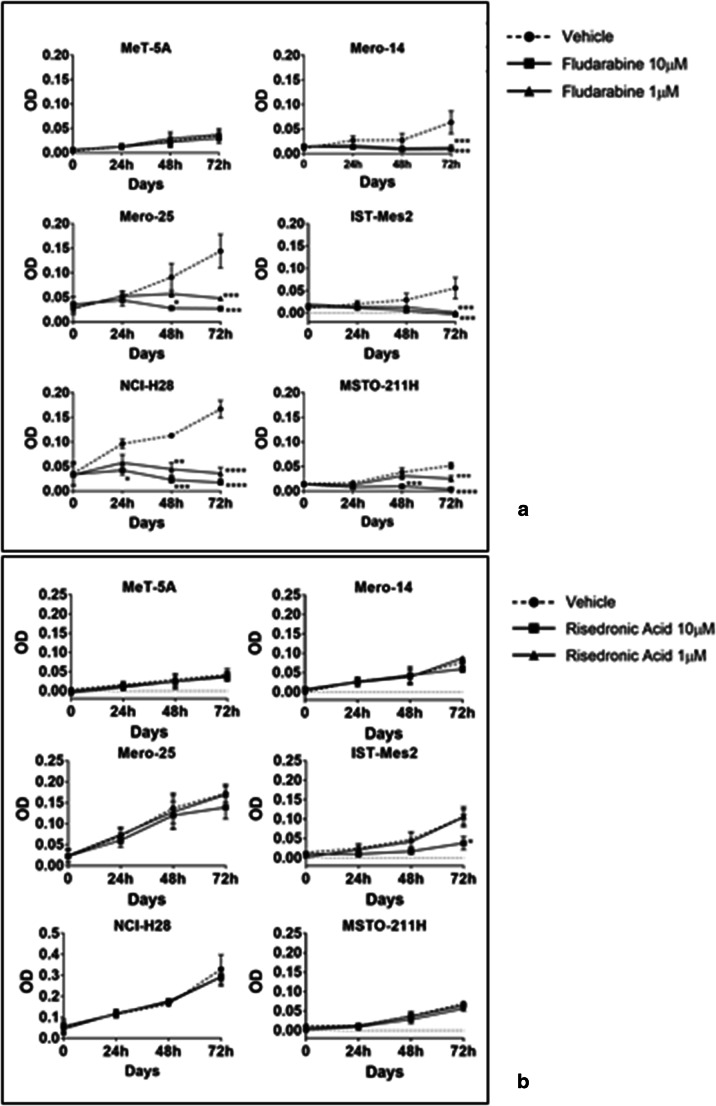
**Optical Density evaluated at 595 nm with the MTT assay for the assessment of cell proliferation of MeT-5A and MPM cell lines (Mero-14, Mero-25, IST-Mes2, NCI-H28, and MSTO-211H) after the treatment with 1 and 10 μM of F-araA (A) or RIS (B)** Proliferation rate was measured with the MTT assay at 24, 48, and 72 h after the treatment with either the vehicle (dotted line) or the drug. Data represent mean ± SEM of three independent experiments, each performed in triplicate. Statistical significance between treated and control is indicated by asterisks as follows: *P* < 0.05*; *P* < 0.01**; *P* < 0.001***

To further investigate the potential of F-araA and RIS is MPM therapeutics, we measured their effect on cell proliferation using additional assays, including the MTT assay and colony formation assays. The MTT assay performed over a period of 72 h of growth by using the doses of 1 μM and 10 μM F-araA and RIS. Treatment with F-araA was effective in reducing the proliferation of MPM cell lines, but not the MeT-5A cells. Significantly at 1 μM F-araA, we observed decreased proliferation rates (up to −90%) of all MPM cell lines, statistically significant at 24, 48, and 72 h, when compared with their respective controls (vehicle). Treatment of MeT-5A with F-araA did also not show any reduction of the proliferation using the MTT assay [Fig. 2A]. RIS was not very effective in reducing the proliferation of MeT-5A and MPM cells. At 10 μM only IST-Mes2 showed a statistically significant reduction of proliferation (up to 60%) upon RIS treatment [Fig. 2B].

**Fig. 3:**
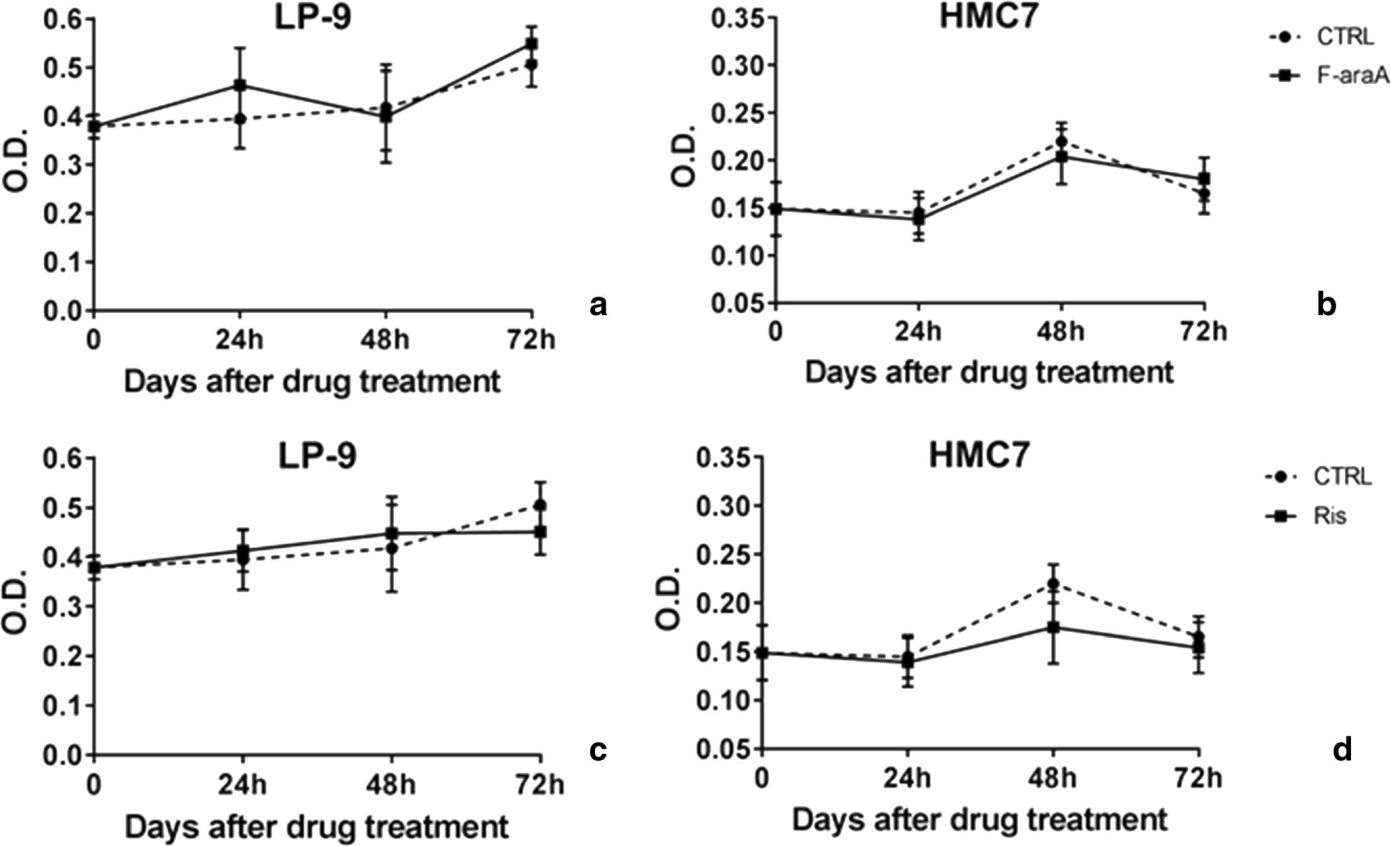
**Proliferation of non-malignant line of mesotheliocytes, as LP-9 and HMC7, after treatment with fludarabine (F-araA) and risedronic acid (RIS)** Proliferation rate was measured 24, 48 and 72 h after either vehicle (dotted lines) or drug treatment (this is day = 0), as F-araA at 1 μM **(A, B)** or RIS at 10 μM **(C, D)**. Data represent mean ± SEM of two independent experiments, each performed in duplicate

We next verified whether additional non-malignant cell lines could validate our results observed in the Met-5A cells. Available models of non-malignant immortalized mesotheliocytes or primary cells from mesothelium are very limited. However, using the LP-9 cell line (a commercially available and non-malignant line of mesotheliocytes) and a primary cell line (HMC7) grown locally from a surgically resected mesothelium of a patient hospitalized for a pneumothorax, we repeated the proliferation assay upon treatment with F-araA and RIS. We observed that the proliferation of these cells was not affected by the administration of both compounds (F-araA at 1 μM and RIS at 10 μM), confirming the observations reported for MeT-5A [Fig. 3].

Overall, we concluded that both compounds, F-araA and RIS were non-toxic upon treatment of non-malignant cell lines and that F-araA is active on MPM cells at, at least, 10-fold lower doses than RIS. When used for the cure of osteoporosis, the hematic peaks of RIS ranged between 1.7 to 3.5 × 10–2 μM in humans [10, 11]. In other words, we observed a mild activity of RIS on MPM cells by employing a dose 300-fold higher than that at the peak. Therefore, we did not proceed with further experimentation using RIS and alternatively focused on investigating the therapeutic potential of F-araA, which we observed to be more cytotoxic in MPM cells at lower doses. Thus, F-araA was further evaluated at 1 and 10 μM for its ability to affect the capacity of isolated cells to form individual colonies using a colony formation (CF) assay. This phenotype is dependent by both the proliferation rate and by the efficiency to trigger a clonal expansion without the presence of neighboring cells that could provide proliferative stimuli. We observed that 1 μM F-araA caused a statistically significant decrease of colonies in the malignant cell lines only [Supplemental Fig. S7]. At 10 μM F-araA, the cytotoxic effect was observed in all cell lines, including MeT-5A. We next evaluated the effects of 1 μM F-araA on the proliferative cell signaling by measuring the activation of ERK1/2 (p44/42 MAPK), i.e. the ratio between the phosphorylated form (pERK) and the total (tERK). At 48 h after 1 μM F-araA treatment, we observed that MeT-5A cells had a similar pERK/tERK ratio than the vehicle, while all MPM cell lines showed a marked decrease, confirming the inhibitory effects of F-araA on MPM cells observed with the proliferation assays [Supplemental Fig. S8].

### Evaluation of putative mechanisms of growth inhibition of MPM cell lines by F-araA

**Fig. 4:**
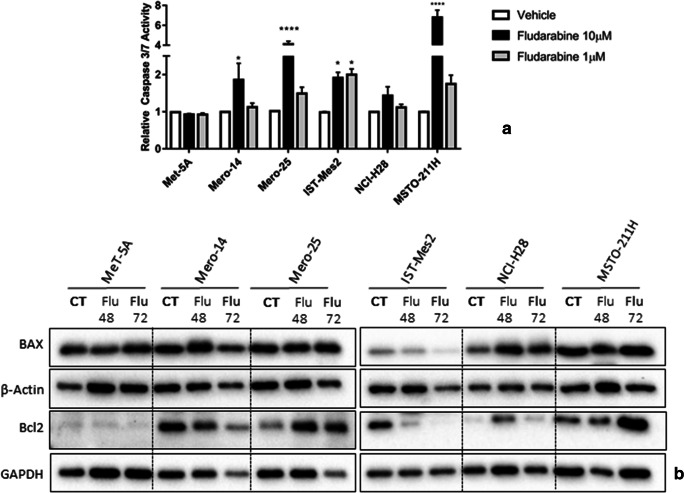
**Apoptosis markers in MeT-5A and MPM cells (as Mero-14, Mero-25, IST-Mes2, NCI-H28, and MSTO-211H) after the treatment with fludarabine (F-araA) (A)** Caspase activities measured in MeT-5A and MPM cells 24 h after treatment with either vehicle (DMSO) or two different concentrations of F-araA, 10 μM and 1 μM. Data represent the mean ± SEM from three independent experiments. Statistical significance between treatment and vehicle is indicated as P < 0.05*, P < 0.01**, and P < 0.001***. **(B)** Western blot analysis of BAX and Bcl2, at 48 or 72 h after a treatment with F-araA at 1 μM. An amount of 10 μg of proteins, obtained from cell lysates, was employed. GAPDH or β-Actin were used as protein loading control

We evaluated several hypotheses for assessing the mechanisms by which F-araA could be active on the MPM cell panel. Firstly, we investigated whether a reduced number of proliferative MPM cells was due to caspase activation, indirectly suggesting the involvement of apoptosis. Upon analysis, however, at 1 μM, overall, the activation of caspases-3 and -7 was found weak in all cell lines with the exception of IST-Mes2 [Fig. 4A]. The western blot showed also that the expression of Bax and Bcl2 was not affected by 1 μM of F-araA at 48 and 72 h after treatment (again with the same exception of IST-Mes2) [Fig. 4B]. Overall, the response of the cell lines in terms of apoptosis markers, did not correlate with the response to the viability and proliferation assays. Thus, we hypothesized that apoptosis is not playing an important role in the inhibitory action of F-araA at 1 μM in our MPM cell panel. However, there could be some exceptions (i.e. IST-Mes2 cells) where apoptosis could be induced. At 10 μM the compound is likely toxic via unspecific mechanisms and an induction of caspases −3 and − 7 is observed in MPM but not in MeT-5A cells [Fig. 4A].

**Fig. 5:**
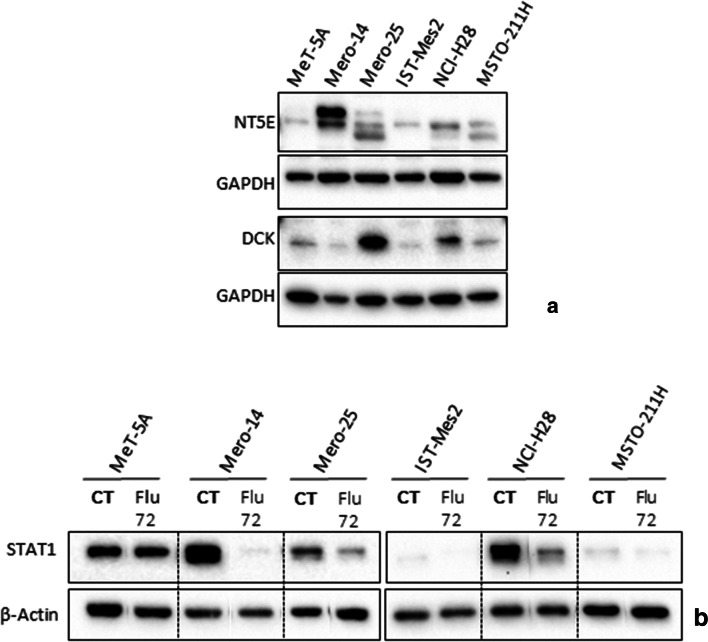
**Fludarabine (F-araA) treatment: Examination of DCK, NT5E, and STAT1, in non-malignant MeT-5A and MPM cells (A)** Expression of NT5E and DCK, the main enzyme involved in the biotransformation of F-araA, at the basal level. **(B)** Inhibitory effects on STAT1 protein expression induced by 72 h treatment with F-araA (1 μM). An amount of 10 μg of proteins, obtained from cell lysates, was employed. GAPDH or β-Actin were used as protein loading control

Another investigation was carried out in order to evaluate whether the sensitivity to F-araA could be ascribed to the expression of NT5E and DCK (encoded by *NT5E* and *DCK* genes) the two main enzymes involved in F-araA biotransformation. However, we measured the basal protein expression by western blot analysis and no correlation was found. In fact, cells highly responsive to F-araA, such as IST-Mes2 and MSTO-211H, showed low expression of NT5E and DCK, to a similar extent to that measured in the non-responsive MeT-5A cell line [Fig. 5A]. In addition, we verified whether the response to F-araA could be related to genetic polymorphisms in *NT5E* and *DCK*, thus we genotyped rs2295890 (G > C) within *NT5E* and rs11544786 (C > T) within *DCK*. However, no correlation was observed. In fact, all the cell lines employed in these experiments were homozygous for the common allele. We next determined whether, the treatment with F-araA caused a down-regulation of the expression of the transcription factor, Signal transducer and activator of transcription 1, (STAT1) in MPM cells. In fact, a specific depletion of STAT1 has been described in various malignant cell lines including human lymphocytes, natural killer cells, breast, colon carcinoma, and neuroblastoma upon F-araA treatment [12]. Here, we confirmed such an effect, in the malignant Mero-14, Mero-25, and NCI-H28 cell lines, STAT1 expression was decreased most significantly at 72 h after F-araA treatment. Significantly, the expression levels of STAT1 were unaffected in MeT-5A cells upon similar F-araA treatment, confirming the lack of effects of F-araA in this cell line. IST-Mes2 and MSTO-211H had a very low basal STAT1 expression and therefore were not informative [Fig. 5B].

**Fig. 6:**
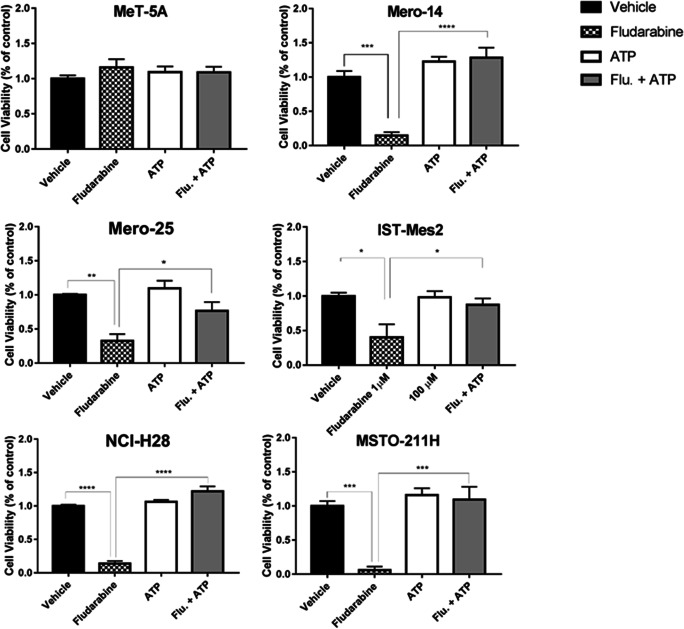
**ATP-mediate restoration of fludarabine viability in MPM and MeT-5A cells** The panel of cells were treated with the vehicle (black bar), F-araA (black dotted bar), ATP (white bar) or with the combination of F-araA and ATP (grey bar). The viability was assessed 72 h after treatment. Error bars are SEM of three different experiments. Statistical significance between treatment and vehicle is indicated by asterisks as follows: P < 0.05*, P < 0.01**, and P < 0.001***

Finally, we investigated whether the action of F-araA could be mediated through the inhibition of the enzyme ribonucleotide reductase, resulting in the depletion of the dNTP pool, as previously seen in other studies. Thus, we carried out cell viability assays where the treatment of 1 μM F-araA was administered together with 50 μM of ATP. In agreement with this known mechanism of F-araA, the co-administration of ATP completely restored the viability of the MPM cells to the levels of vehicle-treated cells (Mero-14, *P* < 0,0001; Mero-25, *P* = 0.029; IST-Mes2, *P* = 0.0422; NCI-H28, *P* < 0.0001; MSTO-211H, *P* = 0.0005) [Fig. 6].

## Discussion

Following a high-throughput drug repositioning screen of an FDA-approved drug library of 1170 compounds at a single dose of 10 μM, we found that a panel of malignant MPM cells, but not MeT-5A, were highly responsive to F-araA and RIS, two molecules scarcely previously investigated in the context of MPM. F-araA belongs to the family of purine analogues and it is already widely employed in the treatment of hematological malignancies, such as leukemia and lymphomas [13–16]. BPs are mainly employed for the treatment of numerous bone disorders, as osteoporosis, hypercalcemia and Paget’s disease [17]. However, they exhibit also antitumor activity, efficiently affecting both the tumor cells and the surrounding microenvironment, as reported in several recent preclinical and clinical studies [18–20]. In particular, zoledronic acid is used with positive outcomes for breast, prostate, and non-small cell lung cancers [21–23] and it was found active in vitro and in vivo against MPM cells [24, 25].

Cell viability following F-araA and RIS treatment was assayed over a range of doses scaled up to 100 μM and compared also with related compounds such as carmofur, cladribine, clofarabine, trifluorothymidine, alendronic acid, ibandronic acid, oxethazaine, and cytarabine, gemcitabine, and zoledronic acid (the last three known to be active against MPM cells). F-araA and RIS showed to be the most active on MPM cells, with the least toxicity for non-malignant Met-5A cells. We extended our investigation on an additional four malignant (two were primary cells grown locally from surgical specimens) and two non-malignant cell lines, confirming that both compounds were not cytotoxic for non-malignant cells and that RIS is less active than F-araA on MPM cells. The toxicity of F-araA has been thoroughly evaluated in humans and this drug obtained the approval as therapeutic agent for the cure of B cell chronic lymphocytic leukaemia. Interestingly, the daily hematic peak dose of 1.33 μM of F-araA for chemotherapies lasting 28-days is tolerated in patients affected by this malignancy [26]. This dosage was of the same order of magnitude of that detected here as active against MPM cell lines. In our hands, RIS showed mildly positive results on MPM cells at doses approximately 300-fold higher than those of the hematic peaks measured in humans when RIS is administered for the cure of osteoporosis. Thus, we decided to concentrate our investigation on F-araA, as the more promising hit compound for a potential use as a chemotherapeutic agent for MPM [10, 11]. F-araA was active at 1 μM also in the CF assay on MPM cells, while a non-significant reduction of CF was observed for MeT-5A. The observed effects were likely not due to the induction of apoptosis due to lack of caspase activation at 1 μM, but should be ascribed to the inhibition of proliferation, confirmed also by the reduced ratio pERK/tERK observed in MPM cells, but not in non-malignant cells. At 10 μM all the cells (including MeT-5A) demonstrated a reduced ability to form colonies. The viability of MeT-5A cells started to be affected at 10 μM F-araA and it is likely that this is due to the increased treatment conditions applied in the CF assay (i.e. repeated treatments of F-araA every 48 h for 14 days), in comparison to the proliferation assays. Significantly, F-araA has been approved for its use in humans and it is well tolerated. Therefore, the fact that non-malignant cell lines were responsive to F-araA in the CF assay at 10 μM should not prevent the consideration of F-araA as a potential drug for use in the therapy of MPM. Furthermore, at 1 μM this compound was clearly more effective on malignant than non-malignant cells.

We verified whether the differential sensitivity to F-araA in MPM cells could be due to an increased activity of NT5E or a reduced activity of DCK. In fact, F-araA is formulated as 5′-monophosphate-nucleotide, in order to enhance its solubility for intravenous infusion when used in vivo. This adenine analogue acts as a “prodrug” and upon its administration, it is converted metabolically through dephosphorylation (by NT5E) to the antimetabolite F-araA, able to enter into cells. DCK then triggers the activation, consisting of three consecutive phosphorylation steps that will convert F-araA into the main active metabolite, the 5′-triphosphate (F-araATP), necessary to exert the cytotoxic activity. Changes in these enzymatic activities are often observed in cells unresponsive to deoxynucleoside analogues, being the most common mechanisms of resistance [27, 28]. However, we observed that the differential responses within our panel of cell lines were not correlated with the expression of NT5E or DCK, nor to specific functional genetic polymorphisms within these gene loci [29].

Significantly, we also confirmed that F-araA could act on MPM cells with mechanisms similar to those reported for other diseases. In fact, as expected, we observed that F-araA could repress the expression of STAT1. This mechanism has been well described in chronic lymphocytic leukaemia [12, 30, 31], neointimal hyperplasia [32], steroid-induced avascular necrosis of the femoral head [33, 34], and vascular disease [35]. F-araA targets STAT1, a well-known component of the Janus kinase JAK/STAT signaling cascade acting as a tumor suppressor [36]. An in silico study showed that it could occur by competition of F-araA with the highly conserved Src homology 2 (SH2) domain of STAT1, a site necessary for STAT1 homodimerization and consequent nuclear translocation [35, 37]. The lack of nuclear STAT1 causes a decreased *STAT1* gene expression by a self-regulatory loop, ending in mRNA depletion and a further reduction of STAT1 at the protein level [12]. STAT1 inhibition by F-araA prevents cells from responding to factors that mediate their activity through STAT1, in particular the type I-III interferons [38], various growth factors (EGF, PDGF), and cytokines (interleukin 21, 27 and 35) [31, 36, 39]. Moreover, we showed that also in MPM cells F-araA acts as antimetabolite. It has structural similarity with the endogenous deoxyadenosine 5′-triphosphate (dATP) and it can be incorporated into DNA and RNA causing inhibition of the enzymes involved in nucleic acid synthesis and repair [40]. Targets of F-araATP comprise DNA polymerases α, β, γ and ε, DNA primase and ligase, and ribonucleotide reductase, whose inhibition results in a depleted dNTP pool [41]. According to this mechanism, we confirmed that supplementation with ATP completely restored the cell viability, despite the presence of F-araA.

In conclusion, we have identified F-araA as a candidate drug deserving further investigation, following in vivo experiments. Since its analogues (i.e. gemcitabine and cytarabine) have been already assayed in clinical trials on MPM (with limited results) and these compounds in our hands showed a lower activity than F-araA, clinical trials on F-araA would be strongly justified. There is the hope that F-araA could represent a novel weapon to be used in combination with current chemotherapeutic agents in the fight against this incurable disease.

## Supplementary Information

ESM 1 (PDF 886 kb)

## Abbreviations

BP: Bisphosphonate
CF: Colony Formation
CI: Combination Index
dATP: deoxyadenosine 5′-triphosphate
DCK: Deoxycytidine Kinase
DMSO: Dimethyl Sulfoxide
DR: Drug Repositioning
F-araA: 9-β-D-arabinosyl-2-fluoroadenine, or fludarabine
MPM: Malignant Pleural Mesothelioma
MTT: (3-(4, 5-dimethylthiazolyl-2)-2,5-diphenyltetrazolium bromide)
NT5E: Ecto-5′-Nucleotidase
RIS: Risedronic Acid
SNP: Single Nucleotide Polymorphisms
SRB: Sulphorodamine B
STAT1: Signal transducer and activator of transcription 1
SV40: Simian Virus 40
