# Supplementary material for: Drug-repositioning screening identified fludarabine and risedronic acid as potential therapeutic compounds for malignant pleural mesothelioma
Source: Invest New Drugs. 2020 Dec 9;39(3):644–57. doi: 10.1007/s10637-020-01040-y (PMC8068714; doi:10.1007/s10637-020-01040-y)

Supplementary Data

Identification of fludarabine and risedronic acid as active compounds in malignant pleural mesothelioma cell lines.

Irene Dell'Anno [1], Sarah A. Martin [2], Marcella Barbarino[3, 4], Alessandra Melani [1], Roberto Silvestri [1], Maria Bottaro [3], Elisa Paolicchi [1], Alda Corrado [1], Monica Cipollini [1], Ombretta Melaiu [1,5], Antonio Giordano [3, 4], Luca Luzzi [6], Federica Gemignani [1,*] and Stefano Landi[1].

[1] Department of Biology, Genetic Unit, University of Pisa, 56126 Pisa, Italy; irene.dellanno@biologia.unipi.it (I.D.); alessandra-29@hotmail.it (A.M.); r.silvestri17@gmail.com (R.S.); eli.paolicchi@gmail.com (E.P.); corradoalda@gmail.com (A.C.); monica.cipollini@unipi.it (M.C.); ombretta.melaiu@unipi.it (O.M.); federica.gemignani@unipi.it (F.G.); stefano.landi@unipi.it (S.L.).

[2] Centre for Molecular Oncology, Barts Cancer Institute, Queen Mary University of London, Charterhouse Square, London, EC1M 6BQ, UK; sarah.martin@qmul.ac.uk (S.M)

[3] Department of Medical Biotechnologies, University of Siena, 53100 Siena, Italy; marcella.barbarino@unisi.it (Marcella Barbarino); president@shro.org (A.G.); mariaeusebia.bottaro@gmail.com (Maria Bottaro)

[4] Sbarro Institute for Cancer Research and Molecular Medicine, Center for Biotechnology, College of Science and Technology, Temple University, Philadelphia, PA 19122, USA.

[5] Immuno-Oncology Laboratory, Department of Paediatric Haematology/Oncology and of Cell and Gene Therapy, Ospedale Pediatrico Bambino Gesù, IRCCS, 00165 Rome, Italy.

[6] Department of Medicine, Surgery and Neurosciences, Siena University Hospital, 53100 Siena, Italy; dr.luca.luzzi@gmail.com

* Correspondence: federica.gemignani@unipi.it; Tel.: +39-050-2211529

Table of Contents

Table S1.....2

Supplementary references.....3

Figure S1.....9

Figure S2.....11

Figure S3.....12

Figure S4.....13

Figure S5.....14

Figure S6.....15

Figure S7.....16

Figure S8.....17

Table S1. Z-scores resulting from the FDA-drug library screening on the non-malignant MeT-5A cells and on Mero-14, Mero-25, IST-Mes2, NCI-H28 and MSTO-211H MPM cell lines. Here, we listed the 28 compounds showing a cytotoxic activity on at least three MPM cell lines and not active on MeT-5A cells. The average ("Mean") and the standard error of the mean ("SEM") from two independent experiments are reported. The last three columns show the presence of previous experimentation on MPM. One plus sign (+) is added for every study (for references see Supplementary references and, for clinical trials, see https://clinicaltrials.gov/). The minus sign (-) reports a lack of studies. **a:** Average of Z-scores of all MPM cell lines; **b:** difference between the average of Z-scores of all MPM cell lines and that of MeT-5A.

	MeT-5A		Mero-14		Mero-25		IST-Mes2		NCI-H28		MSTO-211H				Assayed in		
**Drug Compound**	**Mean**	**SEM**	**Mean**	**SEM**	**Mean**	**SEM**	**Mean**	**SEM**	**Mean**	**SEM**	**Mean**	**SEM**	**MPM[a]**	**Δ[b]**	**in vitro**	**in vivo**	**Clin.Trials**
Chloroxine	-1.65	±0.53	-2.76	±0.14	-2.49	±0.25	-1.89	±1.93	-2.01	±1.46	-1.97	±1.56	-2.22	-0.57	-	-	-
Chlorpromazine	-0.84	±0.44	-2.50	±0.47	-2.05	±1.23	-3.16	±0.69	-0.55	±0.38	-1.26	±0.67	-1.90	-1.06	-	-	-
Dabigatran	-0.83	±0.53	-2.48	±1.01	-2.23	±1.02	-2.03	±1.38	-0.19	±0.21	-1.75	±0.73	-1.74	-0.91	-	-	-
Deferasirox	-0.40	±0.29	-2.43	±0.12	-2.05	±0.10	-2.51	±0.32	-2.47	±0.26	-1.89	±1.34	-2.27	-1.87	+	++	-
Desloratadine	-1.87	±0.30	-2.82	±1.72	-2.20	±1.09	-3.37	±2.23	-3.31	±1.50	-1.86	±0.98	-2.71	-0.84	-	-	-
Dicyclomine HCl	-1.94	±0.26	-2.13	±0.10	-2.09	±0.31	-1.97	±0.09	-1.50	±0.21	0.13	±0.15	-1.51	0.43	-	-	-
Eltrombopag	-1.95	±0.22	-2.86	±0.07	-4.45	±0.22	-4.79	±0.11	-2.13	±0.67	-1.27	±0.93	-3.10	-1.15	-	-	-
Elvitegravir	-1.60	±0.86	0.48	±0.00	-2.38	±0.48	-0.21	±0.01	-2.40	±0.01	-2.26	±0.76	-1.35	0.25	-	-	-
Erlotinib HCl	-0.88	±0.32	-2.72	±0.88	-3.76	±0.45	-2.21	±0.72	-4.59	±1.64	-4.85	±1.49	-3.63	-2.75	>5	+++	+
Ethacridine lactate	-1.87	±1.08	-3.42	±0.07	-2.72	±0.85	-2.54	±0.49	-1.46	±0.73	-0.09	±0.68	-2.05	-0.18	-	-	-
Floxuridine	-1.12	±0.07	-1.50	±0.06	-2.17	±0.00	-2.57	±0.28	-1.57	±0.16	-2.50	±0.90	-2.06	-0.94	-	-	-
Fludarabine	-1.47	±0.08	-0.19	±0.26	-5.55	±0.48	-6.80	±0.47	-8.56	±0.22	-4.04	±0.92	-5.03	-3.56	+	-	-
Imatinib Mesylate	0.85	±0.35	-3.88	±1.23	-3.83	±0.45	-1.59	±0.28	-5.23	3.78	-1.85	±2.19	-3.28	-4.13	+++	>5	++
Imipramine HCl	-1.68	±0.26	-2.11	±0.20	-2.65	±0.32	-2.42	±0.80	-1.09	±0.22	0.04	±0.46	-1.65	0.03	-	-	-
Loperamide HCl	-1.33	±0.71	-3.32	±0.15	-2.42	±0.87	-1.78	±1.05	-3.35	±0.87	-2.08	±0.44	-2.59	-1.26	-	-	-
Manidipine	-1.53	±0.48	-2.87	±0.25	-2.87	±0.29	-3.01	±0.12	-4.49	±0.89	-1.76	±0.73	-3.00	-1.47	-	-	-
Nifuroxazide	0.41	±0.01	-3.36	±0.39	-3.87	±0.95	-0.70	±0.49	-0.04	±0.20	-2.08	±0.49	-2.01	-2.42	-	-	-
Pimozide	-1.00	±0.40	-1.23	±0.84	-1.17	±0.03	-2.26	±1.67	-2.26	±0.46	-2.13	±0.15	-1.81	-0.81	-	-	-
Ponatinib	-0.78	±1.09	-2.31	±0.11	-3.05	±0.71	-3.28	±0.35	-3.98	±0.65	-3.36	±1.05	-3.20	-2.42	+	+	-
Prochlorperazine Dimaleate	-0.23	±0.11	-2.67	±0.27	-2.28	±1.08	-2.32	±0.58	-0.76	±0.28	-1.62	±0.58	-1.93	-1.70	-	+	-
Propafenone	0.65	±0.05	-0.86	±0.55	-1.39	±0.21	-2.56	±0.35	-2.59	±0.22	-2.84	±0.64	-2.05	-2.70	-	-	-
Rimonabant	-1.61	±0.18	-2.30	±0.08	-1.72	±0.20	-2.25	±0.47	-2.95	±0.26	-2.95	±0.46	-2.43	-0.82	-	-	-
Risedronic acid	0.07	±0.26	-0.68	±0.69	-2.99	±0.07	-3.13	±0.07	-4.69	±0.33	-1.97	±1.48	-2.69	-2.76	-	-	-
Sunitinib Malate	-1.62	±2.73	-3.95	±0.09	-3.89	±1.12	-3.80	±1.84	-5.60	3.76	-5.27	±1.07	-4.50	-2.88	++	+++++	+
Tamoxifen Citrate	-0.28	±0.02	-3.05	±0.26	-2.56	±1.15	-2.83	±0.77	-1.35	±0.28	-2.39	±0.00	-2.44	-2.16	++	++	-
Temsirolimus	-1.83	±0.18	-2.57	±0.12	-3.17	±0.74	-4.12	±0.50	-5.40	±0.23	-4.22	±0.86	-3.90	-2.07	+++	++	-
Trifluoperazine 2HCl	-1.89	±1.06	-2.68	±2.12	-4.19	±1.16	-3.23	±2.18	-1.78	±1.40	-1.66	±1.49	-2.71	-0.82	-	-	-
Triflupromazine HCl	-1.84	±0.68	-3.77	±1.00	-2.73	±1.02	-4.56	±1.85	-1.55	±0.09	-1.01	±0.73	-2.72	-0.88	-	-	-

<u>Supplementary references.</u> Scientific publications regarding the compounds reported in Supplementary Table 1 (i.e. compounds showing cytotoxicity on at least three MPM cell lines and no or poor activity on mesothelial MeT-5a) in relation to MPM, with evidences of *in vitro* and/or *in vivo* assessment.

Deferasirox

Nagai H, Okazaki Y, Chew SH, Misawa N, Yasui H and Toyokuni S (2013). Deferasirox induces mesenchymal-epithelial transition in crocidolite-induced mesothelial carcinogenesis in rats. *Cancer prevention research*, 6(11):1222-30. PMID: 24027214.

Jiang L, Chew SH, Nakamura K, Ohara Y, Akatsuka S and Toyokuni S (2016). Dual preventive benefits of iron elimination by desferal in asbestos-induced mesothelial carcinogenesis. *Cancer Science,* 107(7):908-15.PMID: 27088640.

Erlotinib

Liu Z & Klominek J (2004). Inhibition of proliferation, migration, and matrix metalloprotease production in malignant mesothelioma cells by tyrosine kinase inhibitors. *Neoplasia*, 6(6):705-12. PMID: 15720796.

Garland LL, Rankin C, Gandara DR, Rivkin SE, Scott KM, Nagle RB, Klein-Szanto AJ, Testa JR, Altomare DA and Borden EC (2007). Phase II study of erlotinib in patients with malignant pleural mesothelioma: a Southwest Oncology Group Study. *Journal of Clinical Oncology*; 25(17):2406-13. PMID: 17557954.

Jackman DM, Kindler HL, Yeap BY, Fidias P, Salgia R, Lucca J, Morse LK, Ostler PA, Johnson BE and Jänne PA (2008). Erlotinib plus bevacizumab in previously treated patients with malignant pleural mesothelioma. *Cancer*, 113(4):808-14. PMID: 18543326.

Brevet M, Shimizu S, Bott MJ, Shukla N, Zhou Q, Olshen AB, Rusch V and Ladanyi M (2011). Coactivation of receptor tyrosine kinases in malignant mesothelioma as a rationale for combination targeted therapy. *Journal of Thoracic Oncology*, 6(5):864-74. PMID: 21774103.

Barbieri F, Würth R, Favoni RE, Pattarozzi A, Gatti M, Ratto A, Ferrari A, Bajetto A and Florio T (2011). Receptor tyrosine kinase inhibitors and cytotoxic drugs affect pleural mesothelioma cell proliferation: insight into EGFR and ERK1/2 as antitumor targets. *Biochemical Pharmacology*, 82(10):1467-77. PMID: 21787763.

Giovannetti E, Zucali PA, Assaraf YG, Leon LG, Smid K, Alecci C, Giancola F, Destro A, Gianoncelli L, Lorenzi E, Roncalli M, Santoro A and Peters GJ (2011). Preclinical emergence of vandetanib as a potent antitumour agent in mesothelioma: molecular mechanisms underlying its synergistic interaction with pemetrexed and carboplatin. *British Journal of Cancer*, 105(10):1542-53.PMID: 21970874.

Kryeziu K, Jungwirth U, Hoda MA, Ferk F, Knasmüller S, Karnthaler-Benbakka C, Kowol CR, Berger W and Heffeter P (2013). Synergistic anticancer activity of arsenic trioxide with erlotinib is based on inhibition of EGFR-mediated DNA double-strand break repair. *Molecular Cancer Therapeutics,* 12(6):1073-84. PMID: 23548265.

Spring BQ & Kessel D (2018). 3D Culture Models of Malignant Mesothelioma Reveal a Powerful Interplay Between Photodynamic Therapy and Kinase Suppression Offering Hope to Reduce Tumor Recurrence. *Photochemistry and Photobiology*, PMID: 30485439.

Cramer G, Shin M, Hagan S, Katz SI, Simone CB 2nd, Busch TM and Cengel KA (2018). Modeling Epidermal Growth Factor Inhibitor-Mediated Enhancement of Photodynamic Therapy Efficacy Using 3D Mesothelioma Cell Culture. *Photochemistry and Photobiology*, PMID: 30499112.

Fludarabine

Damaraju D, Damaraju VL, Brun M, Mowles D, Kuzma M, Berendt RC, Sawyer MB and Cass CE (2008). Cytotoxic activities of nucleoside and nucleobase analog drugs in malignant mesothelioma: characterization of a novel nucleobase transport activity. *Biochemical Pharmacology*, 75(10):1901-11. PMID: 18371936.

Imatinib Mesylate

Mathy A, Baas P, Dalesio O and van Zandwijk N (2005). Limited efficacy of imatinib mesylate in malignant mesothelioma: a phase II trial. *Lung Cancer*, 50(1):83-6. PMID: 15951053.

Porta C, Mutti L and Tassi G (2007). Negative results of an Italian Group for Mesothelioma (G.I.Me.) pilot study of single-agent imatinib mesylate in malignant pleural mesothelioma. *Cancer Chemotherapy and Pharmacology*, 59(1):149-50. PMID: 16636799.

Bertino P, Porta C, Barbone D, Germano S, Busacca S, Pinato S, Tassi G, Favoni R, Gaudino G and Mutti L (2007). Preliminary data suggestive of a novel translational approach to mesothelioma

treatment: imatinib mesylate with gemcitabine or pemetrexed. *Thorax*, 62(8):690-5. PMID: 17311837.

Ali Y, Lin Y, Gharibo MM, Gounder MK, Stein MN, Lagattuta TF, Egorin MJ, Rubin EH and Poplin EA (2007). Phase I and pharmacokinetic study of imatinib mesylate (Gleevec) and gemcitabine in patients with refractory solid tumors. *Clinical Cancer Research*, 13(19):5876-82. PMID: 17908982.

Bertino P, Piccardi F, Porta C, Favoni R, Cilli M, Mutti L and Gaudino G (2008). Imatinib mesylate enhances therapeutic effects of gemcitabine in human malignant mesothelioma xenografts. *Clinical Cancer Research*, 14(2):541-8. PMID: 18223230.

Saraiya B, Chugh R, Karantza V, Mehnert J, Moss RA, Savkina N, Stein MN, Baker LH, Chenevert T and Poplin EA (2012). Phase I study of gemcitabine, docetaxel and imatinib in refractory and relapsed solid tumors. *Investigational New Drugs*, 30(1):258-65. PMID: 20697775.

Barbieri F, Würth R, Favoni RE, Pattarozzi A, Gatti M, Ratto A, Ferrari A, Bajetto A and Florio T (2011). Receptor tyrosine kinase inhibitors and cytotoxic drugs affect pleural mesothelioma cell proliferation: insight into EGFR and ERK1/2 as antitumor targets. *Biochemical Pharmacology*, 82(10):1467-77. PMID: 21787763.

Tsao AS, Harun N, Lee JJ, Heymach J, Pisters K, Hong WK, Fujimoto J and Wistuba I (2014). Phase I trial of cisplatin, pemetrexed, and imatinib mesylate in chemonaive patients with unresectable malignant pleural mesothelioma. *Clinical Lung Cancer*, 15(3):197-201. PMID: 24492162.

Melaiu O, Catalano C, De Santi C, Cipollini M, Figlioli G, Pellè L, Barone E, Evangelista M, Guazzelli A, Boldrini L, Sensi E, Bonotti A, Foddis R, Cristaudo A, Mutti L, Fontanini G, Gemignani F and Landi S (2017). Inhibition of the platelet-derived growth factor receptor beta (PDGFRB) using gene silencing, crenolanib besylate, or imatinib mesylate hampers the malignant phenotype of mesothelioma cell lines. *Genes Cancer*, 8(1-2):438-452. PMID: 28435517.

Ponatinib

Marek LA, Hinz TK, von Mässenhausen A, Olszewski KA, Kleczko EK, Boehm D, Weiser-Evans MC, Nemenoff RA, Hoffmann H, Warth A, Gozgit JM, Perner S and Heasley LE (2014). Nonamplified FGFR1 is a growth driver in malignant pleural mesothelioma. *Molecular Cancer Research*, 12(10):1460-9. PMID: 24966347.

Prochlorperazine Dimaleate

Sridhar KS, Krishan A, Samy TS, Duncan RC, Sauerteig A, McPhee GV, Auguste ME and Benedetto PW (1994). Phase I and pharmacokinetics studies of prochlorperazine 2-h i.v. infusion as a doxorubicin-efflux blocker. *Cancer Chemotherapy and Pharmacology*, 34(5):377-84. PMID: 8070004.

Risedronic Acid

Wakchoure S, Merrell MA, Aldrich W, Millender-Swain T, Harris KW, Triozzi P and Selander KS (2006). Bisphosphonates inhibit the growth of mesothelioma cells in vitro and in vivo. *Clinical Cancer Research,* 12(9):2862-8. PMID: 16675582.

Sunitinib Maleate

Buckstein R, Meyer RM, Seymour L, Biagi J, Mackay H, Laurie S and Eisenhauer E (2007). Phase II testing of sunitinib: the National Cancer Institute of Canada Clinical Trials Group IND Program Trials IND.182-185. *Current Oncology*, 14(4):154-61. PMID: 17710208.

Laurie SA, Gupta A, Chu Q, Lee CW, Morzycki W, Feld R, Foo AH, Seely J, Goffin JR, Laberge F, Murray N, Rao S, Nicholas G, Laskin J, Reiman T, Sauciuc D and Seymour L (2011). Brief report: a phase II study of sunitinib in malignant pleural mesothelioma. the NCIC Clinical Trials Group. *Journal of Thoracic Oncology*, 6(11):1950-4. PMID: 22005473.

Nowak AK, Millward MJ, Creaney J, Francis RJ, Dick IM, Hasani A, van der Schaaf A, Segal A, Musk AW and Byrne MJ (2012). A phase II study of intermittent sunitinib malate as second-line therapy in progressive malignant pleural mesothelioma. *Journal of Thoracic Oncology*, 7(9):1449-56. PMID: 22895142.

Camidge DR, Blais N, Jonker DJ, Soulières D, Doebele RC, Ruiz-Garcia A, Thall A, Zhang K, Laurie SA, Chao RC and Chow LQ (2013). Sunitinib combined with pemetrexed and cisplatin: results of a phase I dose-escalation and pharmacokinetic study in patients with advanced solid malignancies, with an expanded cohort in non-small cell lung cancer and mesothelioma. Cancer Chemotherapy and Pharmacology, 71(2):307-19. PMID: 23108697.

Blais N, Camidge DR, Jonker DJ, Soulières D, Laurie SA, Diab SG, Ruiz-Garcia A, Thall A, Zhang K, Chao RC and Chow LQ (2013). Sunitinib combined with pemetrexed and carboplatin in patients with advanced solid malignancies--results of a phase I dose-escalation study. *Investigational New Drugs*, 31(6):1487-98. PMID: 23963796.

Uzu M, Sato H, Yamada R, Kashiba T, Shibata Y, Yamaura K and Ueno K (2015). Effect of enhanced expression of connexin 43 on sunitinib-induced cytotoxicity in mesothelioma cells. *Journal Of Pharmacological Sciences*, 128(1):17-26. PMID: 26003083.

Uzu M, Sato H, Shimizu A, Shibata Y, Ueno K and Hisaka A (2017). Connexin 43 enhances Bax activation via JNK activation in sunitinib-induced apoptosis in mesothelioma cells. *Journal Of Pharmacological Sciences,* 134(2):101-107. PMID: 28602541.

Tamoxifen citrate

Pass HW, Temeck BK, Kranda K, Steinberg SM and Pass HI (1995). A phase II trial investigating primary immunochemotherapy for malignant pleural mesothelioma and the feasibility of adjuvant immunochemotherapy after maximal cytoreduction. *Annals of Surgical Oncology*, 2(3):214-20. PMID: 7641017.

Pass HI, Temeck BK, Kranda K, Thomas G, Russo A, Smith P, Friauf W and Steinberg SM (1997). Phase III randomized trial of surgery with or without intraoperative photodynamic therapy and postoperative immunochemotherapy for malignant pleural mesothelioma. *Annals of Surgical Oncology*; 4(8):628-33. PMID: 9416409.

Christodoulou MS, Fokialakis N, Passarella D, García-Argáez AN, Gia OM, Pongratz I, Dalla Via L and Haroutounian SA (2013). Synthesis and biological evaluation of novel tamoxifen analogues. *Bioorganic & Medicinal Chemistry*, 21(14):4120-31. PMID: 23735829.

Jennings CJ, Zainal N, Dahlan IM, Kay EW, Harvey BJ and Thomas W (2016). Tamoxifen Suppresses the Growth of Malignant Pleural Mesothelioma Cells. *Anticancer Research*, 36(11):5905-5913. PMID: 27793915.

Temsirolimus

Hoda MA, Mohamed A, Ghanim B, Filipits M, Hegedus B, Tamura M, Berta J, Kubista B, Dome B, Grusch M, Setinek U, Micksche M, Klepetko W and Berger W (2011). Temsirolimus inhibits malignant pleural mesothelioma growth in vitro and in vivo: synergism with chemotherapy. *Journal of Thoracic Oncology*, 6(5):852-63. PMID: 21358348.

Moriya M, Yamada T, Tamura M, Ishikawa D, Hoda MA, Matsumoto I, Klepetko W, Oda M, Yano S and Watanabe G (2014). Antitumor effect and antiangiogenic potential of the mTOR inhibitor

temsirolimus against malignant pleural mesothelioma. *Oncology Reports*, 31(3):1109-15. PMID: 24378576.

Vazakidou ME, Magkouta S, Moschos C, Psallidas I, Pappas A, Psarra K and Kalomenidis I (2015). Temsirolimus targets multiple hallmarks of cancer to impede mesothelioma growth in vivo. *Respirology*, 20(8):1

Figure S1. Two-dimensional structures of: fludarabine, that acts as a prodrug, and the corresponding active metabolite 9-β-D-arabinosyl-2-fluoroadenine (F-ara-A), exerting the cytotoxic activity; antimetabolites, comprising pyrimidine analogues, as carmofur, cytarabine, gemcitabine and trifluorothymidine, and purine analogues, as cladribine and clofarabine; bisphosphonates, as alendronic acid, ibandronic acid, risedronic acid and zoledronic acid, and oxethazaine.

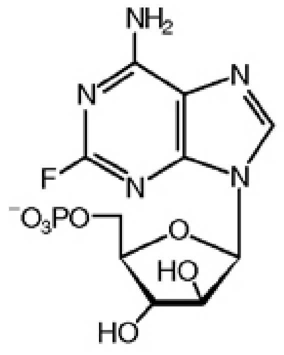

Fludarabine

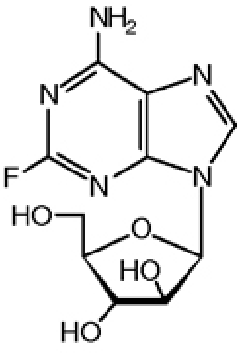

F-ara-A

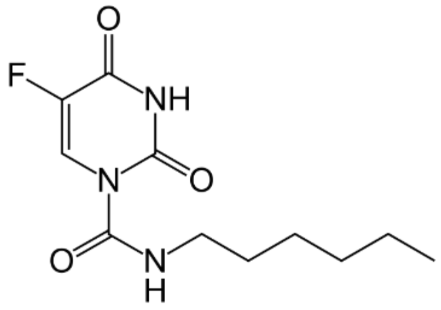

Carmofur

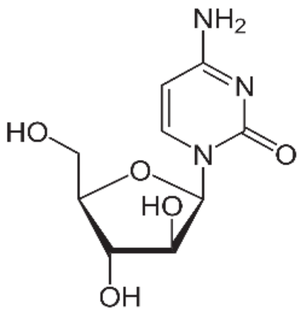

Cytarabine

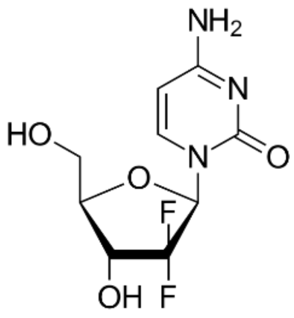

Gemcitabine

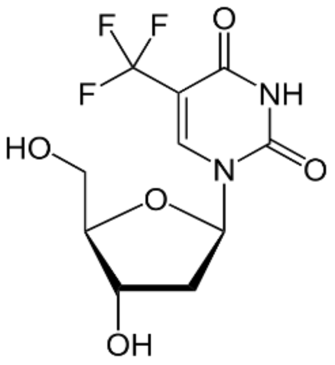

Trifluorothymidine

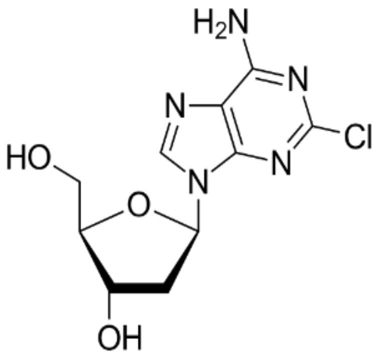

Cladribine

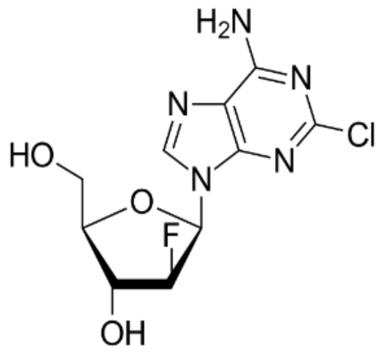

Clofarabine

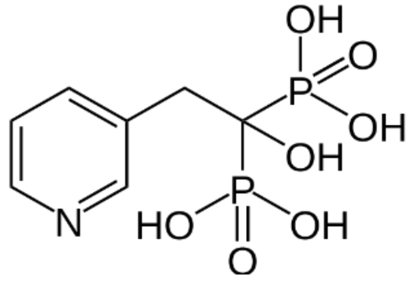

Risedronic Acid

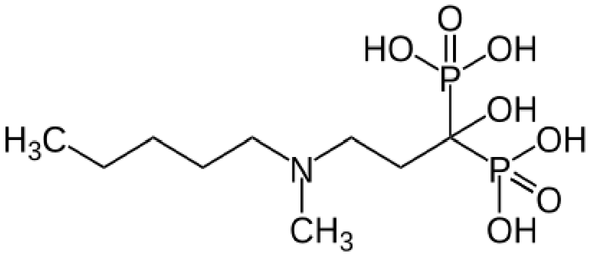

Ibandronic Acid

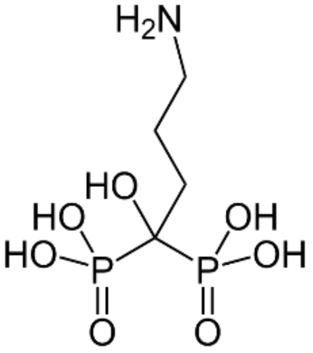

Alendronic Acid

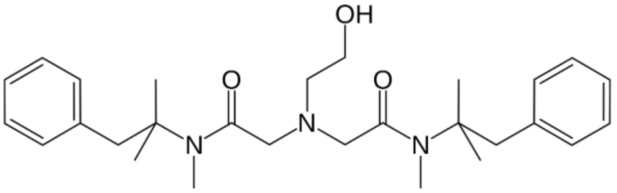

Oxethazaine

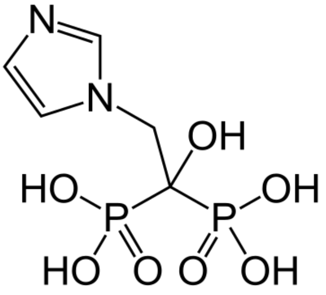

Zoledronic Acid

Figure S2. Cell viability following the treatment with various antimetabolites in non-MPM (MeT-5A, red dotted line) and MPM cells (Mero-14, Mero-25, IST-Mes2, NCI-H28, and MSTO-211H, dark lines). Cells were treated with increasing concentrations (0.1 µM, 1 µM, 10 µM and 100 µM) of the specified compound. Cell viability was measured after four days of treatment using an ATP-based luminescence assay (cell titer assay) and the data represent mean ± SEM of three independent experiments, each performed in triplicate.

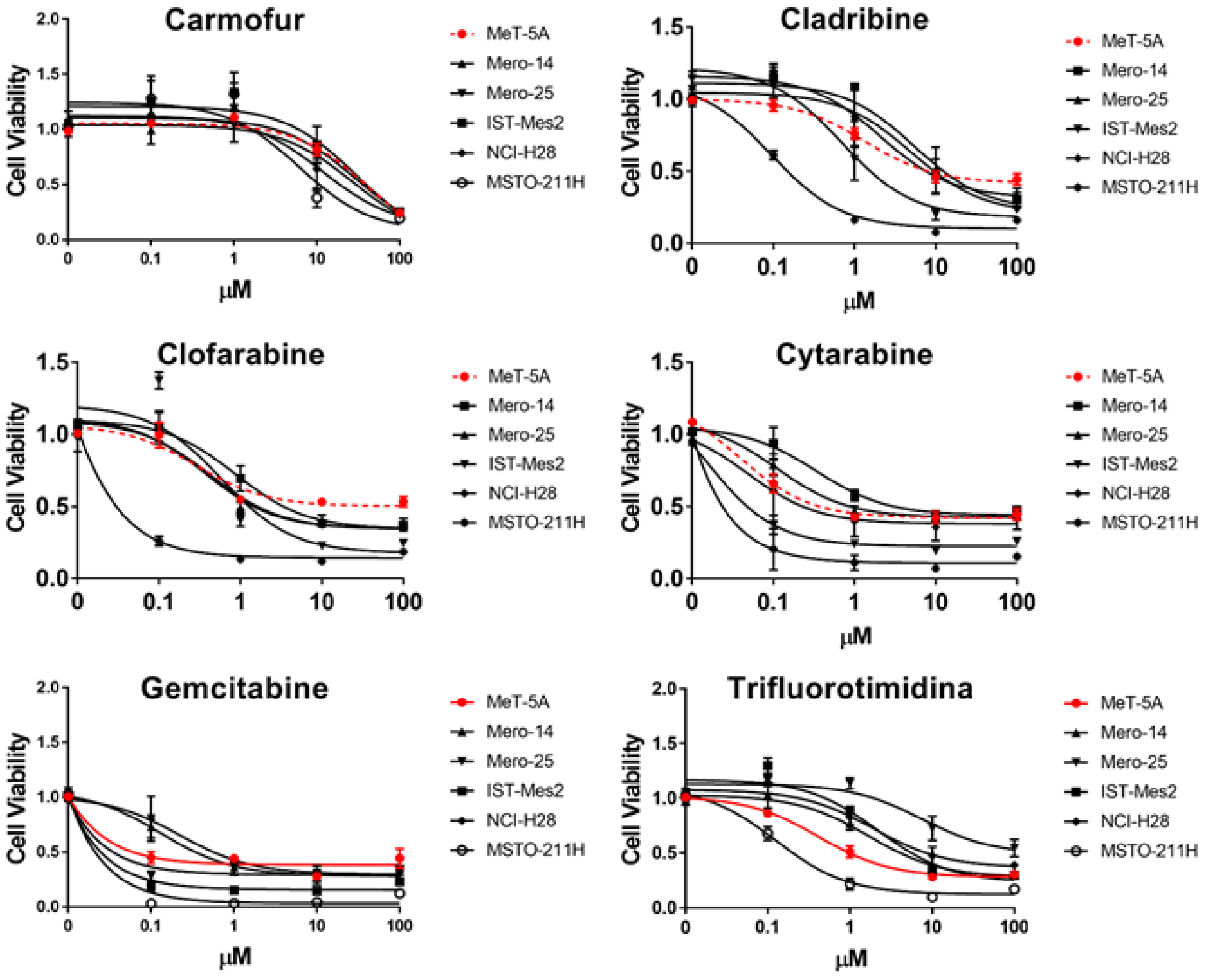

Figure S3. Cell viability following the treatment with bisphosphonates and oxethazaine, in non-MPM (MeT-5A, red dotted line) and MPM cells (Mero-14, Mero-25, IST-Mes2, NCI-H28, and MSTO-211H, dark lines). Cells were treated with increasing concentrations (0.1 µM, 1 µM, 10 µM and 100 µM) of the specified compound. Cell viability was measured after four days of treatment using an ATP-based luminescence assay (cell titer assay). Data represent mean ± SEM of three independent experiments, each performed in triplicate.

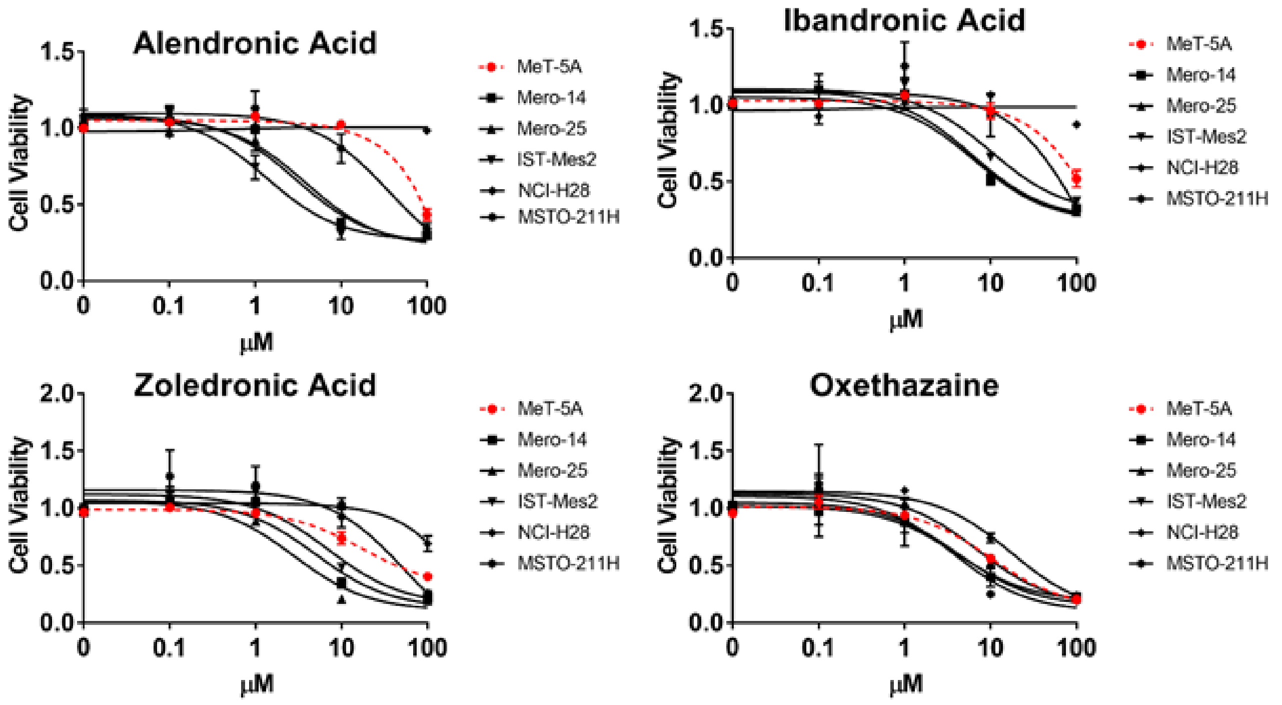

Figure S4. Cell viability of Mero-41 and Ren cell lines following the treatment with fludarabine and risedronic acid. Cell viability was measured after four days of treatment using an ATP-based luminescence assay (cell titer assay). Data represent mean ± SEM of three independent experiments, each performed in triplicate.

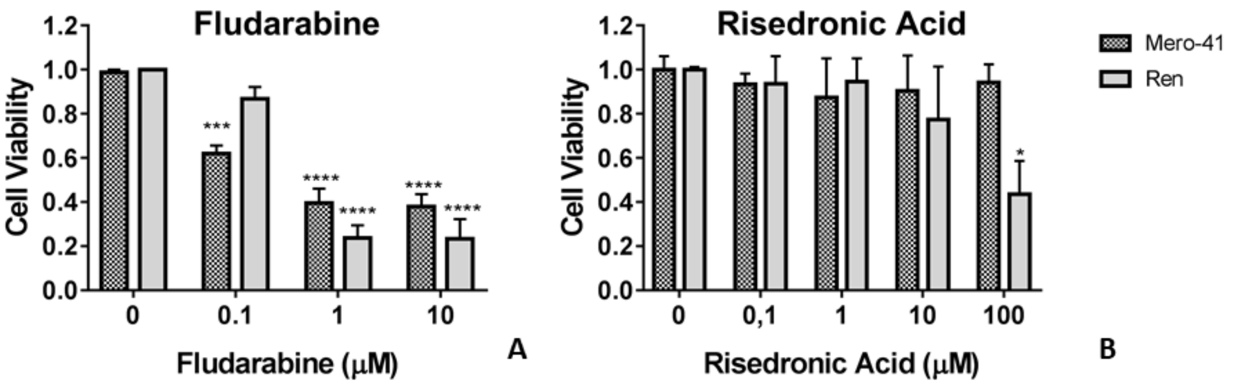

Figure S5. Cytotoxicity produced by fludarabine and risedronic acid in primary cells MMP1 and MMP2. The cell survival was assayed after 72 hours of treatment at the specified concentrations. Data represent mean ± SEM of three independent experiments, each performed in triplicate. Statistical significance is indicated by asterisks (*), where *= P<0.05; **= P<0.01; ***= P<0.001, compared to control treatment.

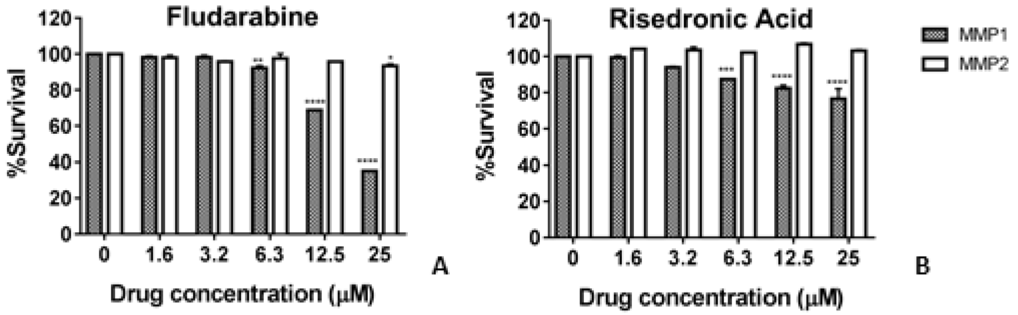

Figure S6. Comparison of the activity of fludarabine (F-araA) and risedronic acid (RIS) with cisplatin and evaluation of possible synergic effects on primary cells cultured from the surgically resected MPM of patient MMP1. (A) Dose-response of the cell survival evaluated after 72 hours of treatment with F-araA (white columns) and cisplatin (black columns) alone or in combination (grey columns). **(B)** Dose-response of the cell survival evaluated after 72 hours of treatment with RIS (white columns) and cisplatin (black columns) alone or in combination (grey columns). Statistical significance for the treatment of F-araA and cisplatin in combination is indicated by asterisks (*), where *= P<0.05; **= P<0.01; ***= P<0.001, compared to F-araA treatment.

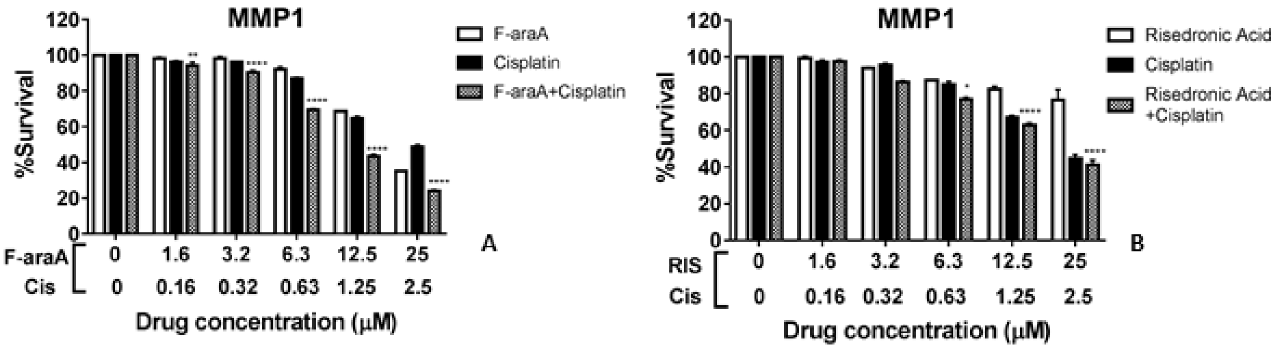

Figure S7. Clonogenicity of mesothelial MeT-5A and a panel of five MPM lines, as Mero-14, Mero-25, IST-Mes2, MSTO-211H and NCI-H28, after treatment with fludarabine (F-araA). (A, C) Representative pictures of colonies in MeT-5A and MPM cell lines, 10 days after treatment with either vehicle (DMSO) or F-araA, at **(A)** 1 µM or **(C)** 10 µM. **(B, D)** Histogram represents number of colonies measured 10 days after vehicle/F-araA **(B)** 1 µM or **(D)** 10 µM treatment, by counting sulphorhodamine-B stained colonies. Error bars are SEM of three different experiments. Error bars are SEM of three different experiments. Statistical significance is indicated by asterisks (*), where *= P<0.05; **= P<0.01; ***= P<0.001, compared to control treatment.

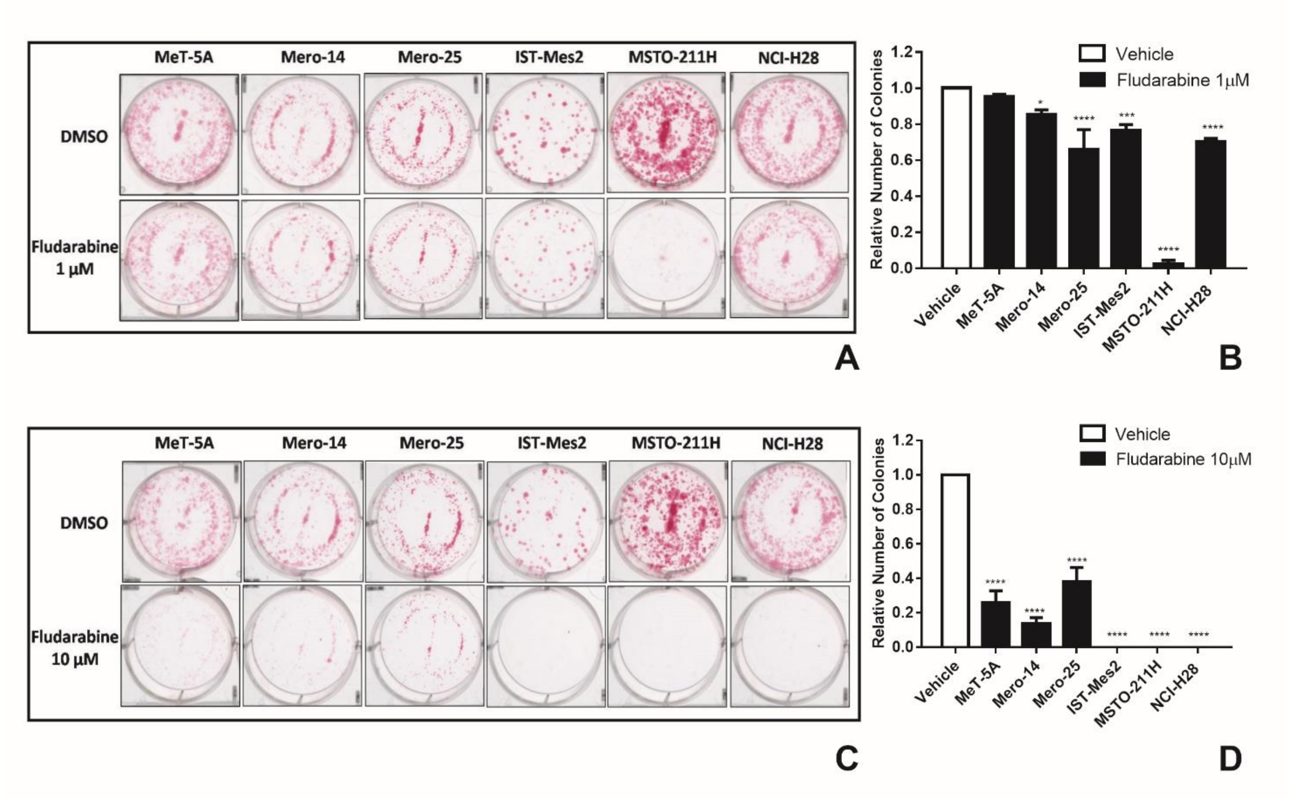

Figure S8. Western blots reporting Erk 1/2 and its phosphorylated form phospho-Erk 1/2 in non-malignant MeT-5A and MPM cells after 48 hours of incubation with fludarabine at 1 µM. An amount of 10 µg of proteins, obtained from cell lysates, was employed. β-Actin was used as protein loading control.

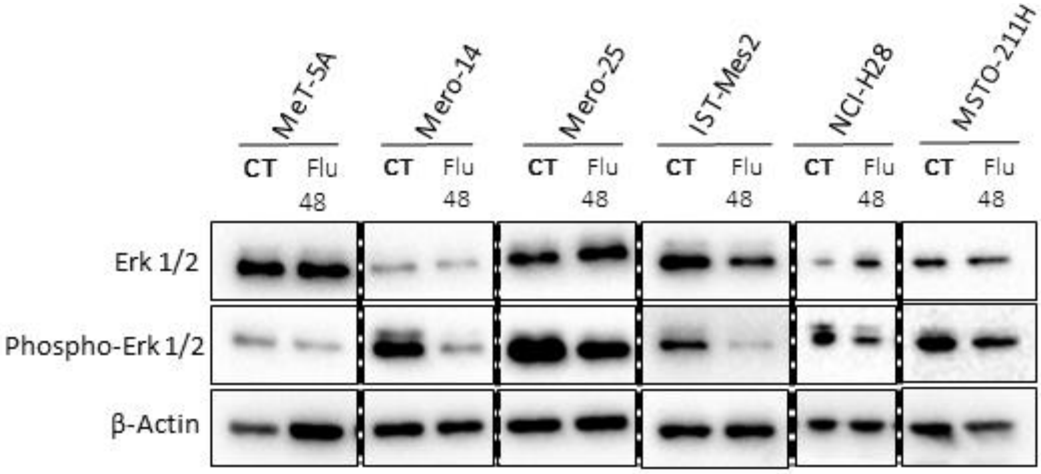

Supplement: Supplementary file 1 — (PDF 886 kb) [file 10637_2020_1040_MOESM1_ESM.pdf]
